# Early OA Stage Like Response Occurs after Dynamic Stretching of Human Synovial Fibroblasts

**DOI:** 10.3390/ijms21113874

**Published:** 2020-05-29

**Authors:** Ute Nazet, Susanne Grässel, Jonathan Jantsch, Peter Proff, Agnes Schröder, Christian Kirschneck

**Affiliations:** 1 Department of Orthodontics, University Medical Centre of Regensburg, D-93053 Regensburg, Germany; peter.proff@ukr.de (P.P.); agnes.schroeder@ukr.de (A.S.); christian.kirschneck@ukr.de (C.K.); 2 Department of Orthopaedic Surgery, Experimental Orthopaedics, Centre for Medical Biotechnology, University of Regensburg, D-93053 Regensburg, Germany; susanne.graessel@ukr.de; 3 Institute of Clinical Microbiology and Hygiene, University Medical Centre of Regensburg, D-93053 Regensburg, Germany; jonathan.jantsch@ukr.de

**Keywords:** osteoarthritis, synovial fibroblasts, mechanical strain, inflammation

## Abstract

As events triggering early osteoarthritis onset can be related to mechanical stress and proinflammatory signaling, we investigated the effect of different mechanical strain protocols on the expression of proinflammatory genes, as well as extracellular matrix remodelling in human synovial fibroblasts. Three distinct models of tensile stretching were applied: static isotropic tensile strain at 0 Hz, 16% tension for 48 h; short-term high-frequency cyclic tension at 1 Hz, 10% tension for 4 h; and dynamic tensile stretching for 48 h, consisting of two blocks of moderate stretching at 0.2 Hz, 2%, advanced stretching at 0.5 Hz, 15%, or a combination of both. General signs of inflammation were present after static isotropic tension, whereas short-term high-frequency cyclic tension showed increased levels of *IL-6* paired with diminished levels of *IL-1β*. Reduced inflammatory effects of *TNF-α*, *IL-6*, and *IL-1β* were observed when exposed to advanced stretching. Long-term tensile strain induced extracellular matrix remodelling at the gene and protein levels. While hyaluronan acid synthesis was increased with static tensile strain, dynamic tensile stretching had a reducing effect. Our study revealed that proinflammatory markers were activated by mechanical strain as seen in static isotropic tension and short-term high-frequency tensile strain, whereas long-term exposure induced extracellular matrix remodelling processes.

## 1. Introduction

Approximately 3.3–3.6% of the worldwide population suffer from osteoarthritis (OA), the most common form of arthritis [1,2]. The etiological factors of developing OA can be classified into two categories: the first comprises factors like age, hormones, obesity, capacity overload, and injuries, while the second, pre-existing joint abnormalities. OA is a complex multi-tissue disease mediated by disturbed homeostasis of cartilage, bone, infrapatellar fat pad and synovium. Beside the role of cartilage in OA etiology, recent studies have identified synovium status as another crucial factor for OA progression [3] and link inflammatory active synovitis with cartilage loss in OA-absent cases [4] (reviewed in reference [5]).

The synovial membrane and the infrapatellar fat pad experience a close proximity, resulting in tissues innervation and close vascularization [6,7]. Beside the synovial membrane, the infrapatellar fat pad has also been shown to be involved in inflammatory processes [8,9,10]. Therefore, it is suggested that the infrapatellar fat pad and the synovial membrane act as functional unit in the pathogenesis of OA. During synovial inflammation, activated inflammatory cells (e.g., T-cells, mast cells, B-cells) infiltrate the synovium [11], and interact with fibroblasts. This induces secretion of synovial-cell-stimulating stress products (e.g., cytokines) and cartilage degradation fragments (e.g., matrix fragments), leading to increased synthesis of chemokines and cytokines as well as prostaglandins, matrix metalloproteinases (MMPs), ADAMTS (a disintegrin and metalloproteinase with thrombospondin motifs), and angiogenic factors [12,13]. The increased expression of proinflammatory and extracellular matrix remodelling factors causes further cartilage degradation, neo-angiogenesis, and synovitis, while activated NF-κB signaling mediates OA-progression-related bone changes [14,15].

A factor inducing early onset of OA can be mechanical stress and its effect on inflammation-mediated NF-κB signaling in articular chondrocytes and synovial cells [16]. Different methods were already published to analyse the effect of mechanical strain on joint tissue in the context of OA progression. An early OA-stage-related status was provoked by shear stress, inducing cyclooxygenase-2 (*COX-2*), dysregulated MMPs, and proinflammatory cytokine production in chondrocytes, while increased interleukin-6 (IL-6) expression mediated immunological processes as well as pro-apoptotic and contra-apoptotic effects on leukocytes [17]. Studies on synovial fibroblasts showed that mechanical stress regulates synovial hyperplasia processes as well as MMP-1, COX-2, and PGE-2 expression [18,19,20]. As histological signs of hyperplasia and elevated levels of COX-2 and PGE-2 are characteristic evidence of OA [5], a correlation between mechanical stress and OA induction is possible. In general, in vitro experiments suffer from inadequate simulation of in vivo situations, especially when mimicking movement and the effect of mechanical strain on cells. For this reason, various protocols and applications are published aiming to overcome this issue [21,22,23,24].

We applied isotropic and dynamic stretching protocols to study both short-term and long-term effects on synovial fibroblasts in promoting OA pathogenesis. In this study, we focused on the effect of different tensile strain protocols on the expression of proinflammatory genes, as well as extracellular matrix remodelling in human synovial fibroblast monocultures.

## 2. Materials and Methods

### 2.1. In Vitro Cell Culture Experimental Setup

Commercially available synovial fibroblasts derived from the knee of healthy, male non-OA patients were obtained from BioIVT (PCD-90-0645; Hicksville, NY, USA) and Cell Applications (408–05a; San Diego, CA, USA). Approximately 35,000 synovial fibroblasts per ml were seeded on a collagen-I-coated 6-well plate (BF-3001C, Dunn Labortechnik, Asbach, Germany) in 2 mL DMEM high glucose per well (D5796, Sigma–Aldrich, Darmstadt, Germany), supplemented with 10% FBS (P30-3306, PAN-Biotech, Aidenbach, Germany), 1% L-glutamine (G7513, Sigma-Aldrich, Darmstadt, Germany), 100 µM ascorbic acid (A8960, Sigma-Aldrich, Darmstadt, Germany), and 1% antibiotics/antimycotics (A5955, Sigma-Aldrich, Darmstadt, Germany) and preincubated under standard cell culture conditions (37 °C, 5% CO_2_, water-saturated) for 24 h. Subsequently, different static or cyclic tensile strain protocols were applied. For cell culture experiments, only cells ranging from passages 3 to 7 were used.

**Figure 1:**
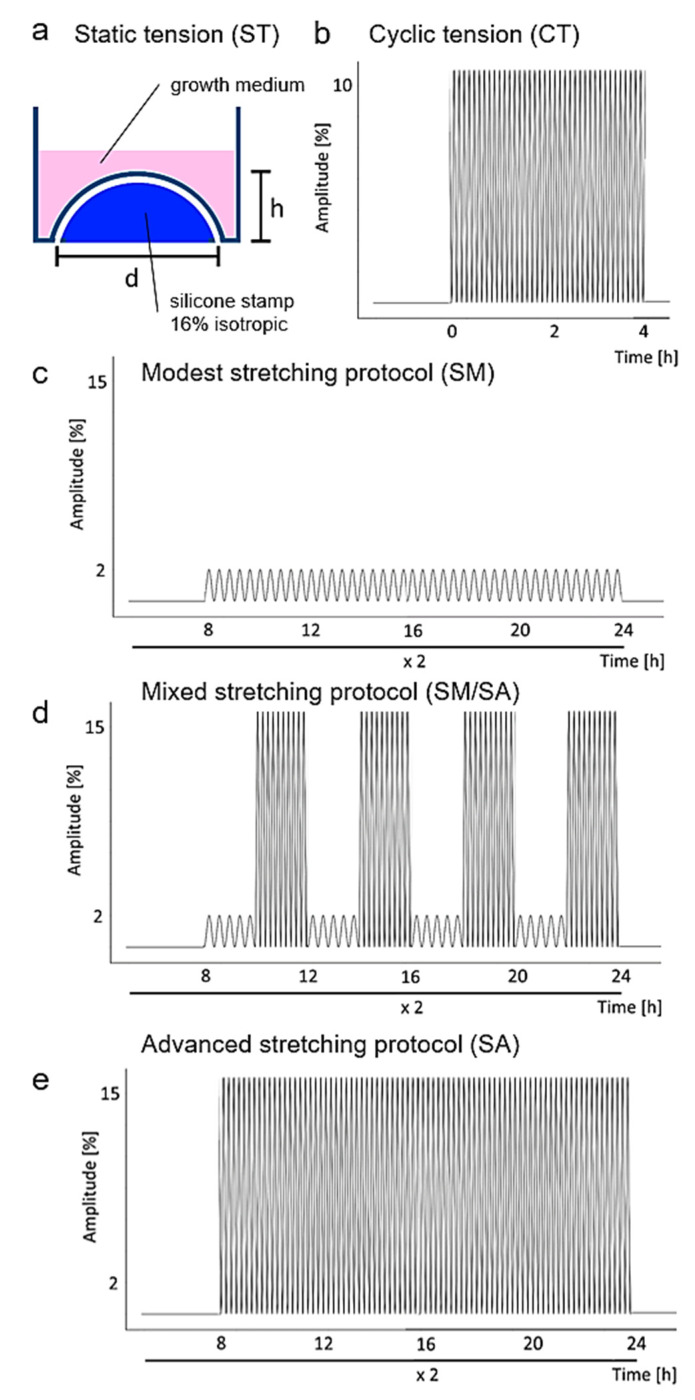
Schematic presentation of the investigated strain profiles. (**a**) Isotropic static tension (ST) of synovial fibroblasts with 16% tensile strain for 48 h. (**b**) Short cyclic tension (CT) with 0–10% elongation, 0.5 Hz for at least 4 h. (**c**) Modest stretching protocol (SM) consisting of 8 h without mechanical strain, followed by 0–2% elongation, 0.2 Hz for 16 h. (**d**) Mixed stretching protocol (SM/SA) consisting of 8 h without mechanical strain, followed by four repetitions of alternate 2 h SM protocol (2% elongation, 0.2 Hz) and 2 h advanced stretching (SA) protocol (15% elongation, 0.5 Hz). (**e**) Advanced stretching protocol (SA) consisting of 8 h without mechanical strain, followed by 0–15% elongation, 0.5 Hz for 16 h. SM, SM/SA and SA protocols were applied for at least 48 h. Control cultures were cultivated under the same culture conditions.

The static isotropic tensile strain (ST, Figure 1a) protocol was adapted from Nazet et al. [21]:

• 1st group (controls): synovial fibroblasts under standard cell culture conditions for a total of 48 h;
• 2nd group: synovial fibroblasts exposed to isotropic static tension of 16% for 48 h. For static tension custom-made isotropic silicon stamps were introduced from the bottom into the flexible surface of the bioflex plates, leading to a surface increase of 16%.

The short-term high-frequency cyclic tension (CT, Figure 1b) protocol was adapted from Muschter et al. [22]:

• 1st group (controls): synovial fibroblasts under standard cell culture conditions for a total of 28 h (consisting of 24 h preincubation and 4 h under normal cell culture conditions);
• 2nd group: synovial fibroblasts exposed to cyclic tension for 4 h (10% tension at a frequency of 1 Hz). For the cyclic tension set-ups short-term high-frequency and dynamic cyclic tensile stretching, a custom-made, cyclic cell-stretching machine consisting of a 6-well fitting slot and 6 stamps, which are simultaneously elongated and retracted according to a previously compiled script, was used. By elongation of the stamps with defined height, operator-defined stretching of the flexible surface with a defined frequency can be obtained.

The dynamic tensile stretching protocol was adapted from Lohberger et al. [23], consisting of four groups:

• 1st group (controls): synovial fibroblasts incubated under standard cell culture conditions for a total of 72 h;
• 2nd group: synovial fibroblasts exposed to a modest stretching (SM, Figure 1c) protocol consisting of cyclic tension for 16 h (2% tension by a frequency of 0.2 Hz) two times including a break of 8 h between the two tension setups;
• 3rd group: synovial fibroblasts exposed to a mixed stretching protocol (SM/SA, Figure 1d) consisting of cyclic tension for 16 h (four repetitions of 2 h 2% tension by a frequency of 0.2 Hz and 2 h 15% tension by a frequency of 0.5 Hz), two times, including a break of 8 h between the two tension setups;
• 4th group: synovial fibroblasts exposed to an advanced stretching protocol (SA, Figure 1e) consisting of cyclic tension for 16 h (15% tension by a frequency of 0.5 Hz), two times, including a break of 8 h between the two tension setups.

### 2.2. Assessment of Cell Number

After the appropriate stretching protocol was applied, cell culture supernatant was removed, and synovial fibroblasts were scraped off the bioflex membrane in 1 mL PBS to determine the cell number. Cells were counted using a Beckman Coulter Counter Z2™ (Beckman Coulter GmbH, Krefeld, Germany) according to the manufacturer’s instructions.

### 2.3. Assessment of Cytotoxicity via LDH Assay

We performed lactate dehydrogenase (LDH) assays (04744926001, Roche, Mannheim, Germany) using respective cell supernatants according to the manufacturer’s instructions. Briefly, we mixed 50 µL of the supernatant with 50 µL LDH solution (freshly prepared of 22 µL catalyst mixed with 1 mL dye) and incubated for 30 min at room temperature in the dark. Afterwards, we added 25 µL stop solution and measured absorbance at 490 nm and 690 nm with an ELISA reader (Multiscan GO Microplate Spectrophotometer, Thermo Fisher Scientific, Waltham, MA, USA).

### 2.4. RNA Isolation and cDNA Synthesis

For gene expression analysis, RNA was isolated from cell pellets using 0.5 mL peqGOLD TriFast™ (072319-30, PEQLAB, Erlangen, Germany) per well, and processed according to the manufacturer’s instructions. The resulting RNA pellet was reconstituted in nuclease-free water (T143, Carl Roth, Karlsruhe, Germany), and after photometrical adsorption measurements (280 nm and 260 nm; NanoDrop, Implen, Munic, Germany), cDNA synthesis was done according to a previous published protocol [25]. Therefore, 0.1 µg RNA was substituted with 2 µL 5x M-MLV-buffer (M1701, Promega, Madison, WI, USA), 0.5 µL dNTP mix (L785.1/2, Carl-Roth, Karlsruhe, Germany), 0.5 µL RNase inhibitor (40U; EO0381, Thermo Fisher Scientific, Waltham, MA, USA), 0.5 µL oligo-dT18 primer (0.1nmol; SO131, Thermo Fisher Scientific, Waltham, MA, USA), 0.5 µL M-MLV reverse transcriptase (M170B, Promega, Madison, WI, USA) and replenished to 10 µL total volume with nuclease-free water (T143, Carl-Roth, Karlsruhe, Germany). After 60 min incubation at 37 °C and 2 min at 95 °C, samples were diluted to a final concentration of 1 ng/µL.

### 2.5. Real-Time Quantitative RT-qPCR and Relative Gene Expression

Gene expression analysis was performed using SYBR^®^Green JumpStart™ Taq ReadyMix™ (S4438, Sigma-Aldrich, Steinheim, Germany) and 1 ng/µL cDNA sample as described before [21,26]. The sample (1.5 µL cDNA) was mixed with SYBR^®^Green JumpStart™ Taq ReadyMix™ (7.5 µL), nuclease-free water (5.25 µL) and specific primers (0.375 µL, 10 pmol; primer synthesis and purification done by Eurofins MWG Operon LLC, Huntsville; High Purity Salt Free Purification HPSF^®^) on ice in 96-well PCR plates (TW-MT, 712282; Biozym Scientific GmbH, Hessisch Oldendorf, Germany), sealed for qPCR reaction (BZO Seal Filmcover; 712350; Biozym Scientific GmbH, Hessisch Oldendorf, Germany). The PCR program consisted of 5 min incubation (95 °C) and 45 cycles of 10 s denaturation (95 °C), 8 s annealing (60 °C) and 8 s extension (72 °C), followed by a final melting curve. The RT-qPCR reactions were performed in a Mastercycler^®^ ep realplex-S thermocycler (Eppendorf AG, Hamburg, Germany) and the used oligonucleotides were designed based on the gene sequences achieved from the nucleotide database NCBI (GeneBank, National Centre for Biotechnology Information) and validated for the absence of secondary structures, self and cross dimers, as well as primer efficiency and specificity (Table 1).

Based on Cq values, the relative gene expression was analysed as 2^−∆Cq^ with ∆Cq = Cq (target gene) − Cq (HK) [27,28], while HK was assessed by the square root of the sum of Cq of the housekeeper genes *RPLP0* and *EEF1A1* [25].

### 2.6. Assessment of Total Collagen Content in Cell Culture Supernatant

Total collagen was assessed with the supernatant of each well according to the manufacturers’ instructions (K218-100, Total Collagen Assay Kit Colorimetric, Biovision, Milpitas, CA, USA). By adding 12 M HCl and incubation at 120 °C (3 h), acidic hydrolysis takes place and hydroxyprolines are formed. Adjacent to this, the formed hydroxyprolines react in an oxidative reaction to a chromophore with specific absorbance at 560 nm, correlating to the sample’s collagen content.

### 2.7. Determination of Hyaluronic Acid Fragment Content in Cell Culture Supernatant

Hyaluronic acid fragment content was validated after glycosaminoglycans were isolated from 1 mL supernatant. Supernatants were freeze-dried and dissolved in 300 µL H_2_O_dd_, substituted with 900 µL ethanol (96%, denatured) and incubated overnight at −20 °C. After centrifugation (5000 rpm, 5 min, 4 °C), supernatants were discarded and the pellet was dissolved in 300 µL 100 mM ammoniumacetat (T872, Carl-Roth, Karlsruhe, Germany) supplemented with 0.107 U proteinase K (P4850, Sigma-Aldrich, Steinheim, Germany) and incubated for 2 h on a rotary shaker (55 °C, 700 rpm), followed by proteinase K inactivation at 100 °C for 5 min. After the substitution of 900 µL ethanol (96%, denatured), samples were stored overnight at −20 °C and centrifuged (5000 rpm, 5 min, 4 °C) the next day. The supernatant was removed, and the pellet dissolved in 50 µL H_2_O_dd_. For HPLC-based hyaluronic acid analysis, 10 µL were mixed with 2 µL chondroitinase ABC (C3667, Sigma-Aldrich, Steinheim, Germany) in 18 µL buffer (0.1 M Tris; 0.15 M sodiumacetat; pH 8.0) and digested overnight at 37 °C. The reaction was blocked by boiling the solution for 1 min. We analyzed the unsaturated disaccharides generated from hyaluronic acid and chondroitin sulphate after enzymatic treatment of the purified glycosaminoglcyans by strong anion exchange HPLC separation (column: Sphere-Image 80-5 SAX, Knauer, Berlin, Germany) at 232 nm. Isocratic separation was from 0 to 15 min with 10 mM NaH_2_PO_4_, pH4.0 and linear gradient separation was from 15 to 35 min with 10 mM NaH_2_PO_4_, pH 4.0 to 33% 750 mM NaH_2_PO_4_, pH 4.0 (flow rate 1.2 mL/min). HPLC equipment was from Shimazdu.

### 2.8. Statistical Analysis

Prior to statistical analysis, all absolute data values were divided by the respective arithmetic mean of the control group without mechanical strain to obtain normalized data values relative to these controls, set to 1. Statistical analysis was conducted with GraphPad Prism 8 (GraphPad Software, San Diego, CA, USA). Data distribution was tested by Shapiro–Wilk tests. All tests were two-sided and the level of statistical significance was set to *p* ≤ 0.05. For ST and CT analysis, non-parametric Mann–Whitney-U tests for independent variables and a comparison between groups were performed, while for comparison of multiple groups Brown–Forsythe- and Welch-corrected analyses of variance (ANOVA), followed by Games–Howell posthoc tests were performed. In graphs, arithmetic mean and standard errors are shown and data displayed in a scatter dot plot.

## 3. Results

### 3.1. Effects of Different Mhanical Strain Protocols on Cell Number and Cytotoxicity

**Figure 2:**
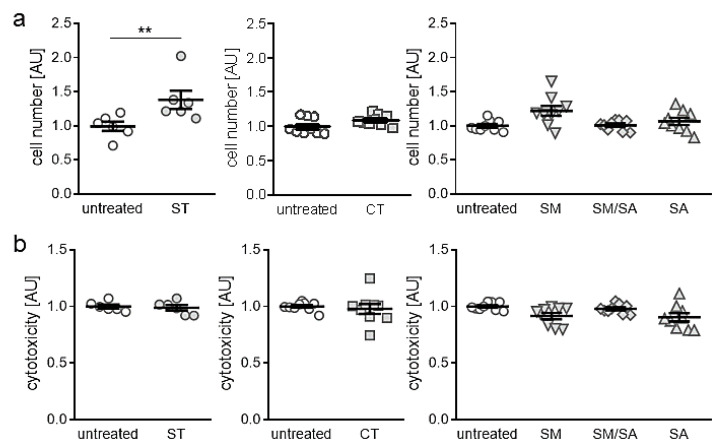
Assessment of cell numbers (**a**) and cytotoxicity (**b**) after application of different tensile strain protocols (explanation see manuscript text). AU: arbitrary units, error bars represent standard error of the mean. *n* > 6; *Statistics:* ST/CT: Mann-Whitney-U tests; SM, SM/SA, SA: Welch-corrected ANOVA with Games-Howell posthoc tests; ** *p* < 0.01.

First, we tested, if the applied tensile strain protocols impact on cell number or induce cytotoxic effects. Beside static isotropic tensile strain, which increased cell number significantly (*p* = 0.006; Figure 2a), we detected no changes in cell number with the short-term high-frequency cyclic tensile treatment (*p* = 0.065) or any of the dynamic tensile stretching protocols (SM: *p* = 0.072., SM/SA: *p* = 0.997, SA: *p* = 0.686, Figure 2a). In accordance with this, we observed no cytotoxic effects of the static (ST: *p* = 0.859), cyclic (CT: *p* = 0.386) or dynamic tensile stretching protocols (SM: *p* = 0.068, SM/SA: *p* = 0.644, SA: *p* = 0.114, Figure 2b), as no enhanced lactate dehydrogenase (LDH) release was present in the cell culture supernatant.

### 3.2. Effects of Various Tensile Strain Protocols on the Expression of Proinflammatory Genes

**Figure 3:**
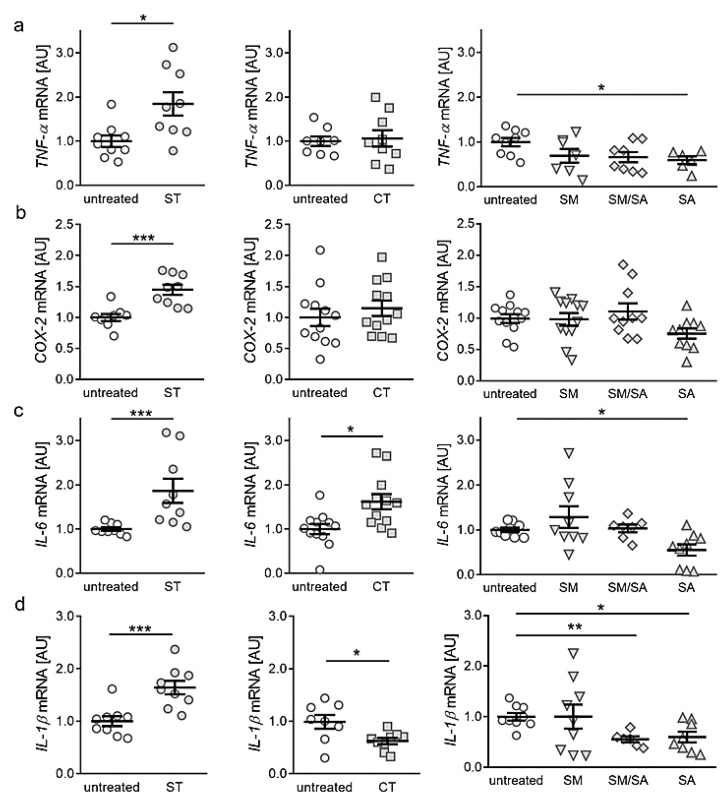
Expression profiles for proinflammatory genes *TNF-α* (**a**), *COX-2* (**b**), *IL-6* (**c**) and *IL-1ß* (**d**) after application of different tensile strain protocols (explanation see manuscript text). AU: arbitrary units, error bars represent standard error of the mean. *n* > 6; *Statistics:* ST/CT: Mann-Whitney-U tests; SM, SM/SA, SA: Welch-corrected ANOVA with Games-Howell posthoc tests; * *p* < 0.05, ** *p* < 0.01, *** *p* < 0.001.

Next, we investigated the impact of different strain protocols on the expression of genes with key functions in local and systemic inflammation. Static tensile strain increased gene expression of tumour necrosis factor α (*TNF-α*, *p* = 0.011, Figure 3a) significantly, while short cyclic, modest and mixed stretching protocols did not affect *TNF-α* gene expression (CT: *p* = 0.963, SM: *p* = 0.372, SM/SA: *p* = 0.152; Figure 3a). Advanced stretching even reduced *TNF-α* mRNA in synovial fibroblasts (*p* = 0.033, Figure 3a). Regarding cylcooxygenase-2 (*COX-2*) expression, static isotropic tensile strain increased expression of *COX-2* (*p* < 0.001), whereas none of the tested cyclic protocols showed any effect (Figure 3b). Analysis of interleukin gene expression revealed that the gene expression of interleukin-6 (*IL-6*) was enhanced by static (*p* < 0.001) and short cyclic tensile strain (*p* = 0.007, Figure 3c). In contrast, modest and mixed protocols did not affect *IL-6* gene expression (SM: *p* = 0.661; SM/SA, *p* = 0.985), whereas a significant downregulation was obtained under the impact of the advanced stretching protocol (*p* = 0.033; Figure 3c). Expression of the gene *IL-1ß* was increased upon static tensile strain (*p* < 0.001), while cyclic tensile strain protocols rather reduced *IL-1ß* mRNA (CT: *p* = 0.038; SM/SA: *p* = 0.002, SA: *p* = 0.036, Figure 3d). Only the modest stretching protocol had no effect on *IL-1ß* gene expression (*p* > 0.999; Figure 3d). Based on these findings, our data suggest a rather proinflammatory phenotype after long-term isotropic stretching. In contrast, dynamic stretching protocols seem to prevent the expression of proinflammatory genes in synovial fibroblasts.

### 3.3. Effects of Various Tensile Strain Protocols on Collagen Synthesis

**Figure 4:**
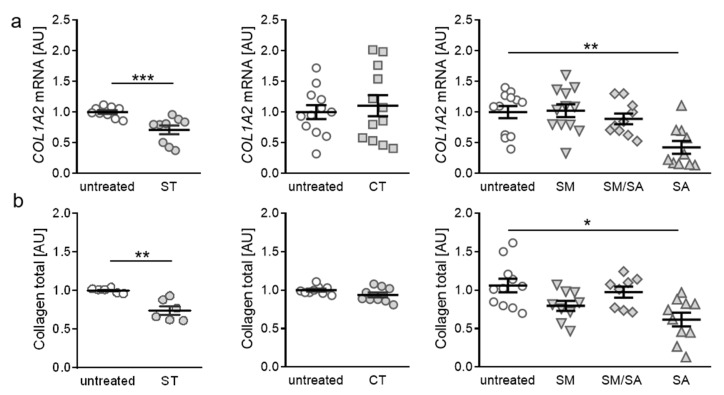
Expression of *COL1A2* (**a**) and total collagen content in cell culture supernatant (**b**) after application of different tensile strain protocols (explanation see manuscript text). AU: arbitrary units, error bars represent standard error of the mean. *n* > 6; *Statistics:* ST/CT: Mann-Whitney-U tests; SM, SM/SA, SA: Welch-corrected ANOVA with Games-Howell posthoc tests; * *p* < 0.05, ** *p* < 0.01, *** *p* < 0.001.

After assessment of proinflammatory gene expression, we focused on the impact of tensile strain stimulation on extracellular matrix remodelling, based on collagen expression. When applying static tensile strain, gene expression of collagen-I-alpha-2 (*COL1A2*) was significantly reduced (*p* < 0.001, Figure 4a). The short-term high-frequency cyclic tension, modest or mixed stretching protocols did not affect *COL1A2* gene expression (CT: *p* = 0.843, SM: *p* > 0.999, SM/SA: *p* = 0.837). In contrast, advanced stretching reduced *COL1A2* gene expression significantly (*p* = 0.004; Figure 4a), indicating that heavy loads lead to impaired collagen synthesis. To further verify our findings, we investigated total collagen content in cell culture supernatant after exposure to different strain protocols. In line with RT-qPCR data, we detected no impact on total collagen content after cyclic tension, modest or mixed stretching (CT: *p* = 0.182, SM: *p* = 0.114, SM/SA: *p* = 0.873; Figure 4b). Both static as well as advanced stretching, however, induced a downregulation of total collagen content in the cell culture supernatant (ST: *p* = 0.002; SA: *p* = 0.011. Figure 4b). This further supports the finding that heavy loads, regardless of static or dynamic tensile strain, influence collagen expression and deposition in synovial fibroblasts.

### 3.4. Effects of Different Tensile Strain Protocols on Hyaluronic Acid

**Figure 5:**
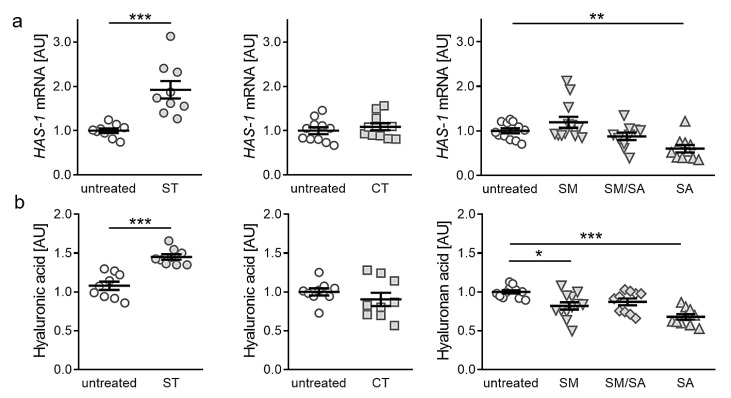
Expression of *HAS-1* (**a**) and hyaluronic acid fraction content in cell culture supernatant (**b**) after application of different tensile strain protocols (explanation see manuscript text). AU: arbitrary units, error bars represent standard error of the mean. *n* > 8; *Statistics:* ST/CT: Mann-Whitney-U tests; SM, SM/SA, SA: Welch-corrected ANOVA with Games-Howell posthoc tests; * *p* < 0.05, ** *p* < 0.01, *** *p* < 0.001.

Finally, we investigated the impact of static or dynamic strain protocols on the expression of hyaluronic acid, as it is a major composition of lubricant joint fluid composition and during OA hyaluronic acid homeostasis is disturbed. Gene expression of hyaluronic acid synthase 1 (*HAS-1*) was significantly enhanced after static tensile strain (*p* < 0.001, Figure 5a). As in collagen synthesis, neither short term cyclic tension nor modest or mixed stretching protocols impacted on *HAS-1* gene expression (CT: *p* = 0.365, SM: *p* = 0.510, SM/SA: *p* = 0.617). In contrast to static tensile strain protocol, advanced stretching resulted in a significantly reduced *HAS-1* gene expression (*p* = 0.005, Figure 5a). To further substantiate our findings, we performed HPLC analysis of detached hyaluronic acid fragments. In line with RT-qPCR data, we observed an increased hyaluronic acid fraction content available in cell culture supernatant, after static tensile strain (*p* < 0.001; Figure 5b). Short term cyclic and mixed stretching protocols did not impact on hyaluronic acid content (CT: *p* = 0.297; SM/SA: *p* = 0.081). Modest and advanced stretching, however, reduced hyaluronic acid content in cell culture supernatant (SM: *p* = 0.013; SA: *p* < 0.001; Figure 5b). In contrast to collagen expression, a beneficial effect of static tensile strain on hyaluronic acid is observable, while effects seen in dynamic agitation of synovial fibroblasts correlate to observed collagen effects.

## 4. Discussion

Synovial fibroblasts (SF) express various recognition receptors, making them capable to sense joint damage as well as invading pathogens [29]. Several stimuli (e.g., cytokines, growth factors, adipokines) are able to activate SF and promote inflammation and joint destruction [30,31].

In order to retrace the effect of mechanical strain and understand OA-related processes, different studies analysed the effect of tensile strain and cyclic mechanical strain on gene expression in the context of OA pathology. We chose two published cyclic tensile strain protocols characterised by a single session of short high-frequency tensile strain and compared it to prolonging static tensile strain and varying cyclic tensile strain. Based on the comparison of the different set-ups, insight into inflammation and extracellular matrix deposition was obtained.

First, analysis of cell proliferation and viability were done and showed no effects of short-term high-frequency cyclic and dynamic stretching on synovial fibroblasts. This served as proof that observed findings in gene expression and extracellular matrix deposition were not caused by a reaction towards apoptotic or cytotoxic effects. Only in static isotropic stretching an increase in cell number was observed. As adherent synovial fibroblasts were used in the experiments, the persistently increased surface by static isotropic stretching most likely offered more space for increased cell proliferation leading to higher cell numbers.

In macrophages cyclic tensile strain increased the expression of mechanotransduction-related NK1R (neurokinin receptor 1) gene, which mediates reduction of cell adhesion presumably by altered SP (substance P) gene and protein expression [22], whereas in chondrocytes mechanical stimulation altered the integrin FAK-MAPK mechanotransduction cascade and activated integrins [23]. Like chondrocytes and macrophages, synovial fibroblasts showed an altered gene expression of *interleukin-6* (*IL-6*), when triggered with mechanical strain. When bruises occur, synovial fluid comprises higher levels of IL-6 and activates an inflammatory response, climaxing in subchondral bone layer changes by dampened type II collagen production [32,33,34]. TNF-α acts synergistically with IL-6 and IL-1β in osteoclast activation [35]. When activated, it diminishes type II collagen synthesis in chondrocytes. Beside this, it induces COX-2, PGE2, and IL-6- synthase, playing an important role in the course of OA [36,37,38]. During OA, the cartilage releases increased amounts of IL-1β, stimulating chondrocytes and promoting inflammation and cartilage destruction [39,40]. In OA pathology increased levels of IL-1β can be detected in the synovial fluid and joint tissue [41,42,43,44]. The proinflammatory marker IL-6 plays a role in the pathophysiology of OA [45]. Increased IL-6 levels can be found in osteoarthritic groups [46], while synovial fluid levels of IL-6 seem not to correlate with OA severity [47]. The application of pressure as mechanical strain has a major impact on gene expression in synovial fibroblasts [48]. Mechanical strain can induce inflammation and can alter extracellular matrix composition. In contrast, application of static tensile strain on fibroblasts of periodontal origin caused an anti-inflammatory reaction [21]. We saw an arthritis-associated inflammation in case of static isotropic tensile strain, as the proinflammatory markers (TNF-α, IL-1β, IL-6, COX-2) were significantly upregulated. By the application of short-term high-frequency tensile strain *IL-6* indicated an inflammatory status similar to macrophages [22]. In contrast, observed downregulation of *IL-1β* indicated absence and damping of inflammation. Beside inflammation induction, IL-6 also acts in innate immune response acquisition, mononuclear cell recruitment and T-cell apoptosis inhibition. Furthermore, IL-6 functions in an anti-inflammatory manner, while activated STAT3-guided signalling supports epithelial cell proliferation and inhibits cell apoptosis [49]. As TNF-α shows no altered gene expression, while IL-1β is downregulated, the induced IL-6 gene expression in the short-term high-frequency tensile strain set-up may be due to activation of regenerative processes in an anti-inflammatory manner [49,50,51]. Contrary to this, beneficial anti-inflammatory effects were achieved when synovial fibroblasts were exposed to dynamic stretching. A correlation with *TNF-α*, which is involved in IL-6 regulation [39,52], was observed for static isotropic stretching and advanced stretching. Our results indicate that short-term high-frequency cyclic tensile strain induced a mechanical stress response, as a significant increase in the inflammatory and innate immune response activating marker *IL-6* could be detected. This concurs with previous findings applying pressure on fibroblasts [24,48]. Interestingly, the application of the dynamic stretching protocol had beneficial effects on *IL-6* expression in synovial fibroblasts, contrary to chondrocytes [23]. Based on findings on chondrocytes exposed to shear stress, an activation of proinflammatory cytokines by COX-2 was assumed [17]. In contrast, we detected no significant changes in *COX-2* gene expression while expression of several proinflammatory genes were altered.

In ongoing OA inflammation processes, chondrocytes switch to a degradative phenotype and activate matrix-degrading proteinase secretion, inducing articular cartilage destruction by secretion of MMPs and ADAMTS [53,54]. Beside these events, catabolic cytokines and chemokines will activate a positive feedback loop, enhancing articular cartilage damage and promoting synthesis of cartilage inflammation, as well as enhancing chondrocyte apoptosis, matrix protein, and collagen degradation. Type I collagen is present in almost all connective tissues and able to stabilize structures with its enormous tensile strength [55]. In the case of cartilage fissure, local tensile stress can build up, promoting chondrocyte phenotype change and type I collagen formation [56]. In late-stage OA, increased levels of type I collagen are present, whereas type II collagen decreases [57]. Our findings indicate that also synovial fibroblasts, originating from the synovium, change gene expression of collagens due to tensile stress. We see a decrease in *COL1A2* expression suggesting extracellular matrix remodelling induced by isotropic and dynamic stretching. In line with these findings, the presence of COL fractions was also diminished by tensile strain.

Hyaluronic acid synthase 1 (HAS-1) is involved in hyaluronic acid (HA) synthesis and plays a major role in joint lubrication [11]. Altered levels of HA are associated with inflammation and degeneration occurring in arthritis [58,59]. A deficiency of HAS-1 is associated with chronic joint inflammation and intra-articular fibrosis [60]. When static isotropic tension was applied to the synovial fibroblasts, increased levels of HA GAGs and HAS-1 were present, similar to observations done in a compressive setup [48]. In contrast, dynamic tensile strain reduced HA GAGs in the cell supernatant and *HAS-1* expression, favouring inflammatory processes.

As the synovium and the infrapatellar fat pad experiences a close proximity to each other, tissue innervation and close vascularization [6,7] enable tissue cross-talks, when molecular imbalances occur. Therefore, when analysing the effect of mechanical strain on synovial fibroblast monoculture metabolism, it is possible that no adequate picture of inflammation induction or changes in extracellular matrix deposition can be observed, as synergistic effects induced by the crosstalk of infrapatellar fat pad are prevented.

## 5. Conclusions

We observed that proinflammatory markers, also involved in OA pathogenesis, are activated in synovial fibroblasts as a reaction to mechanical strain, whereas extracellular matrix-related markers are activated by long-term mechanical strain. Early OA stage similarities in proinflammatory processes and extracellular matrix remodelling could be observed after static isotropic tension and short-term high-frequency tensile strain. In contrast, long-term dynamic stretching promoted anti-inflammatory processes related to extracellular matrix remodelling.

## Figures and Tables

**Table 1:** Reference genes (*EEF1A1*, *RPLP0*) and targets used for RT-qPCR in human synovial fibroblasts.

Gene Symbol	Gene Name	Accession Number	5′-Forward Primer-3′	5′-Reverse Primer-3′
*COL1A2*	collagen, type I, α2	NM_000089.3	AGAAACACGTCTGGCTAGGAG	GCATGAAGGCAAGTTGGGTAG
*COX-2*	cyclooxygenase 2	NM_000963.3	GAGCAGGCAGATGAAATACCAGTC	TGTCACCATAGAGTGCTTCCAAC
*EEF1A1*	eukaryotic translation elongation factor 1	NM_001402.5	CCTGCCTCTCCAGGATGTCTAC	GGAGCAAAGGTGACCACCATAC
*HAS-1*	hyaluronan synthase 1	NM_001523.3	GAGCCTCTTCGCGTACCTG	CCTCCTGGTAGGCGGAGAT
*IL-1β*	interleukin 1β	NM_000576.3	TTCGACACATGGGATAACGAGG	TTTTTGCTGTGAGTCCCGGAG
*IL-6*	interleukin 6	NM_000600.3	TGGCAGAAAACAACCTGAACC	CCTCAAACTCCAAAAGACCAGTG
*RPLP0*	ribosomal protein, large, P0	NM_001002.3	GAAACTCTGCATTCTCGCTTCC	GACTCGTTTGTACCCGTTGATG
*TNF-α*	Tumor necrose factor α	NM_000594.3	GAGGCCAAGCCCTGGTATG	CGGGCCGATTGATCTCAGC
